# Supplementary material for: New insights on patterns of genetic admixture and phylogeographic history in Iberian high mountain populations of midwife toads
Source: PLoS One. 2022 Dec 1;17(12):e0277298. doi: 10.1371/journal.pone.0277298 (PMC9714896; doi:10.1371/journal.pone.0277298)
Supplement: S1 Fig — (a) Geographic location of populations included in each analysis. For population codes see Fig 1. The inset map shows the distribution of the main lineages: orange—ND4 haplogroups E-F (A. almogavarii), yellow—ND4 haplogroup B (A. o. obstetricans), blue—ND4 haplogroup A (A. o. pertinax), red—ND4 haplogroup C (A. o. boscai), green—ND4 haplogroup D (A. o. boscai), black—unclear (adapted from Dufresnes and Martínez-Solano [23]). (b) Markers used in each analysis, with the corresponding number of samples and populations. Effective population sizes were calculated only for populations with ≥ 15 genotyped individuals. In the case of demographic history, only high mountain populations were included in the analysis. (PDF) [file pone.0277298.s001.pdf]

(a)

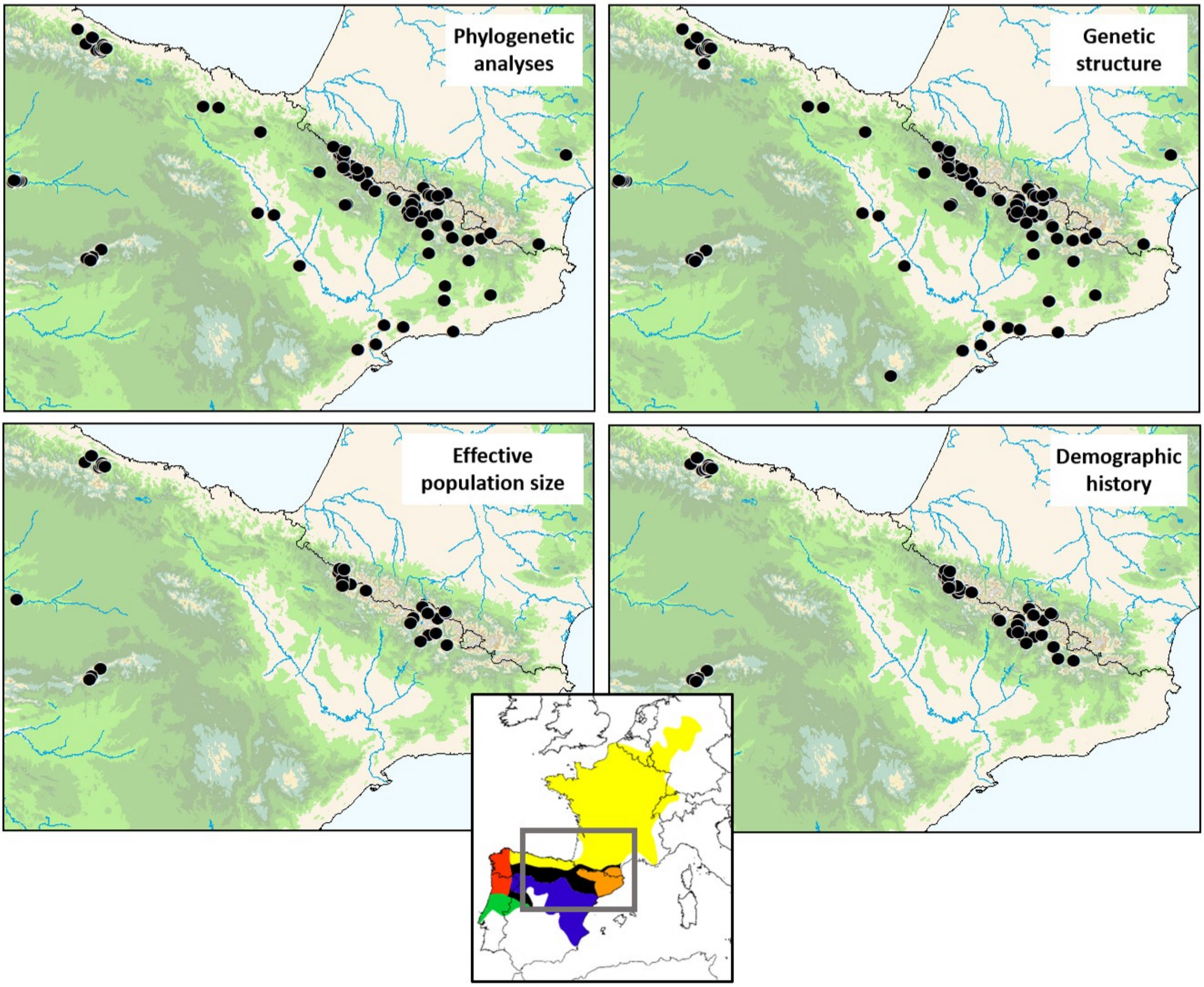

(b)

Type of analysis	Markers used	N Samples/Populations
Phylogenetic analyses	Nuclear (β-fibint7) and mitochondrial (ND4, cyt-*b*, 12S, 16S) gene fragments	β-fibint7: 20/15, ND4: 219/95, cyt-*b*: 40/26, 12S: 42/28, 16S: 40/27
Genetic structure	Microsatellites	878/102
Effective population size	Microsatellites	622/34
Demographic history	Microsatellites + ND4	Microsatellites: 250/55, ND4: 136/55
