# Supplementary figures and images for: New insights on patterns of genetic admixture and phylogeographic history in Iberian high mountain populations of midwife toads
Source: PLoS One. 2022 Dec 1;17(12):e0277298. doi: 10.1371/journal.pone.0277298 (PMC9714896; doi:10.1371/journal.pone.0277298)

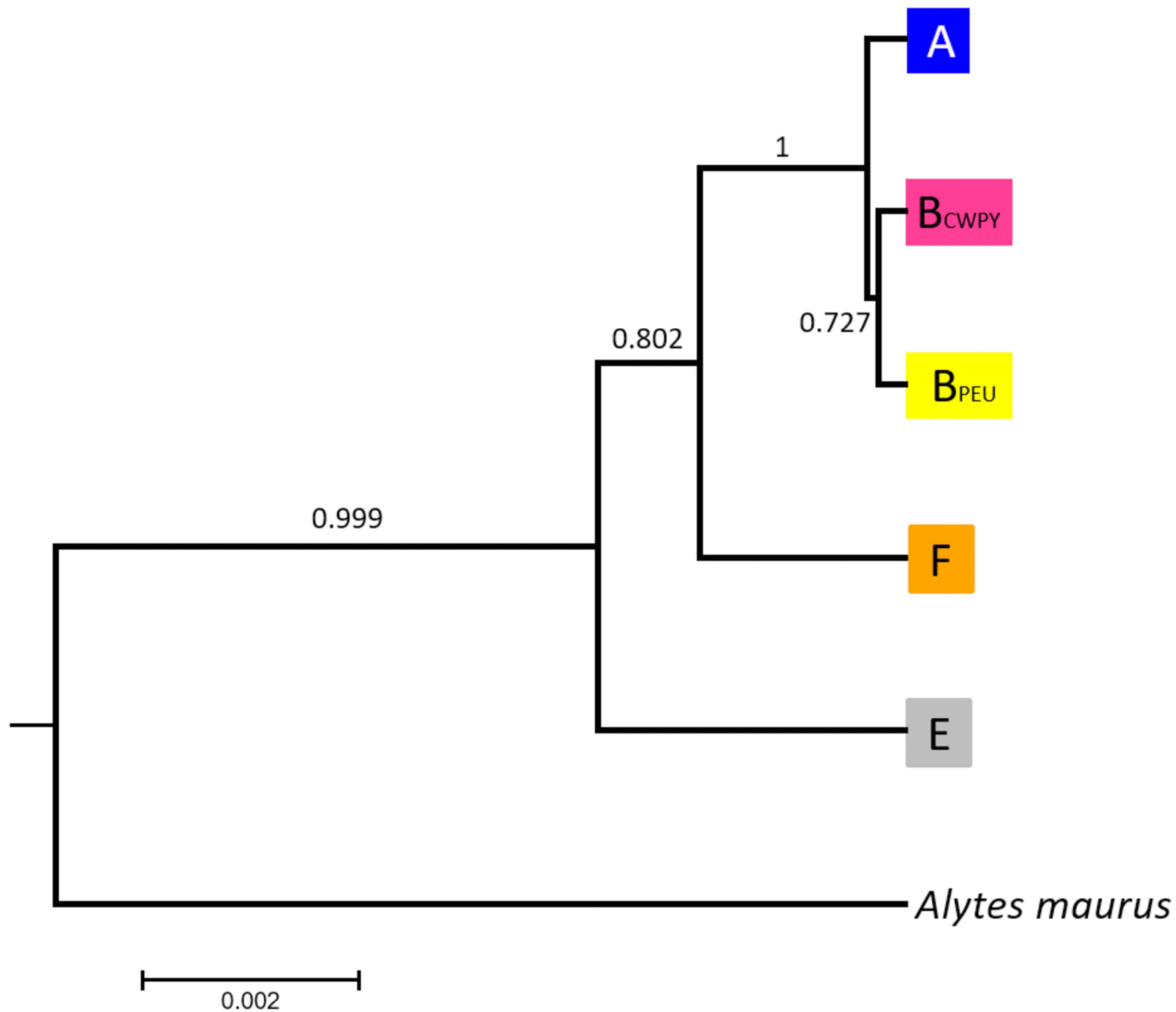

Supplement: S2 Fig — Labels on branch tips correspond to the distinct ND4 haplogroups identified (blue: mtDNA haplogroup A, pink: mtDNA haplogroup B (central-western Pyrenean populations), yellow: mtDNA haplogroup B (Picos de Europa populations), grey: mtDNA haplogroup E, orange: mtDNA haplogroup F). Posterior probabilities of lineage divergence are indicated on branch labels. (PDF) [file pone.0277298.s002.pdf]

(a)

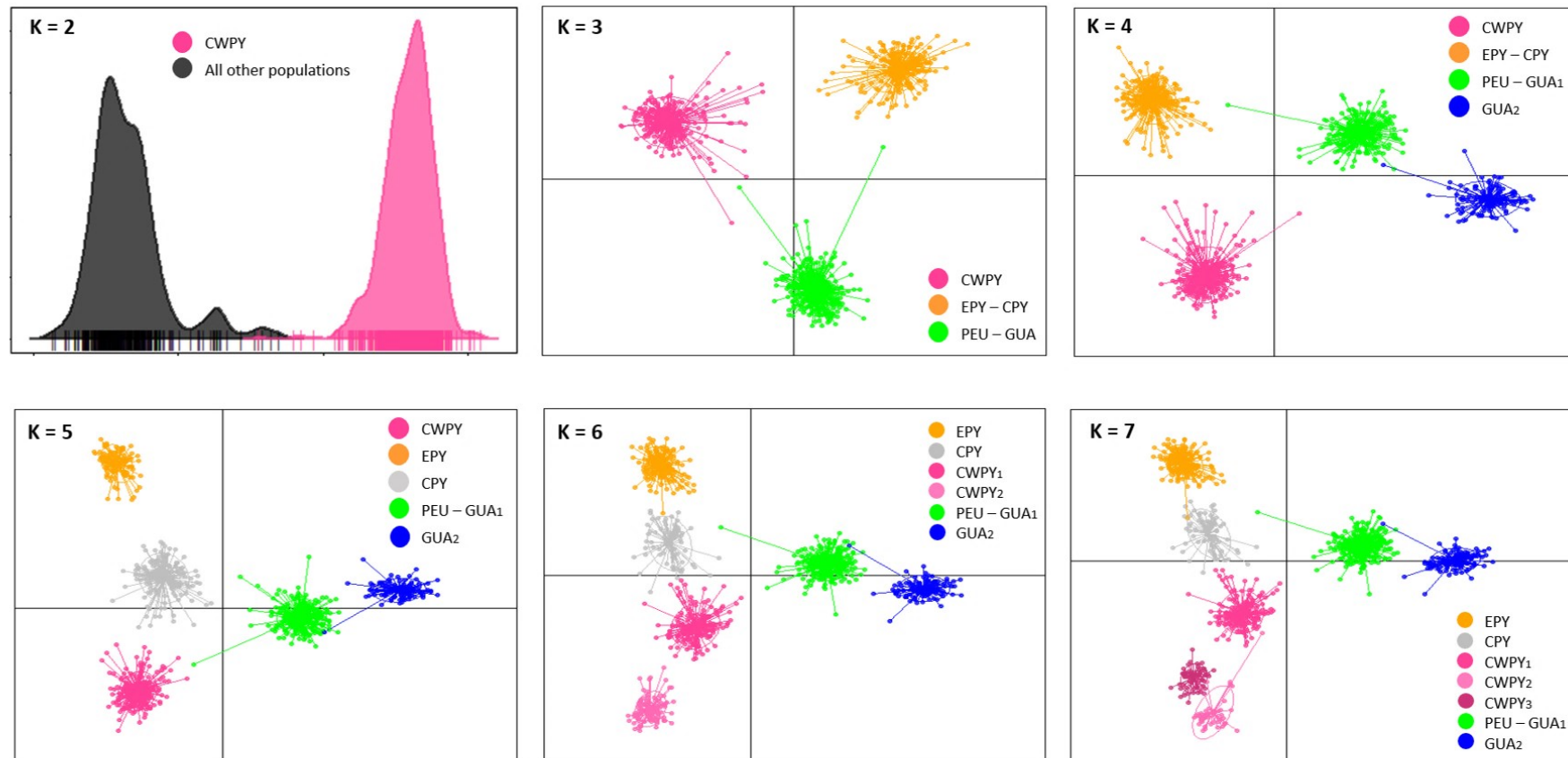

(b)

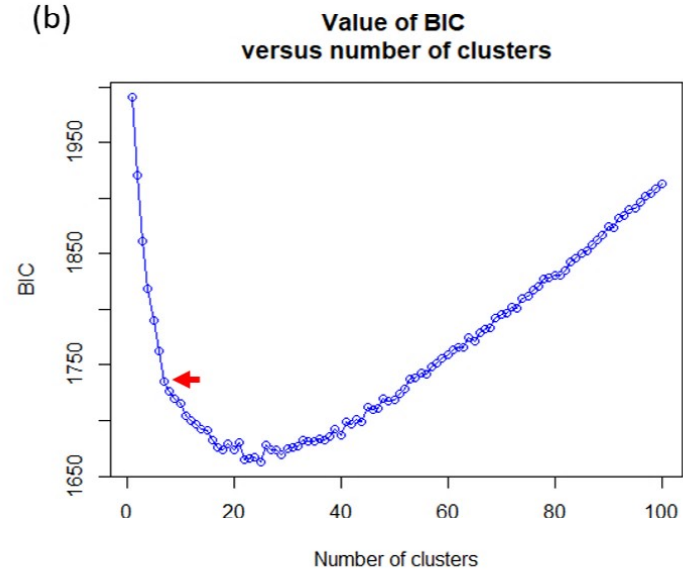

Supplement: S3 Fig — (a) Summary plots for K = 2–7 genetic clusters. At K = 2, genetic clusters are represented as density curves. At K = 3–7, dots represent individuals and genetic clusters are shown as inertia ellipses. Legend labels indicate the different genetic clusters: eastern Pyrenees (EPY, orange), central Pyrenees (CPY, grey), central-western Pyrenees (CWPY, pink), Picos de Europa mountains (PEU, yellow), and Guadarrama Mountain Range (GUA, blue). (b) Distribution of BIC (Bayesian Information Criterion) values according to the number of clusters. The red arrow indicates the number of clusters chosen for DAPC analysis. The optimal number of clusters was assessed using the find.clusters function and determined as the K value above which BIC (Bayesian Information Criterion) values decreased substantially. (PDF) [file pone.0277298.s003.pdf]

(a)

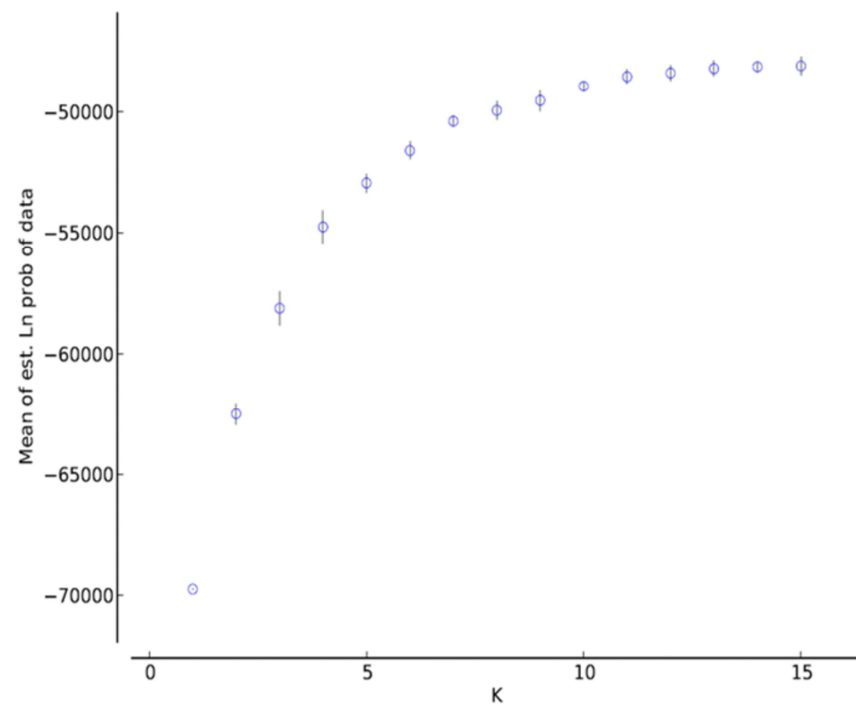

(b)

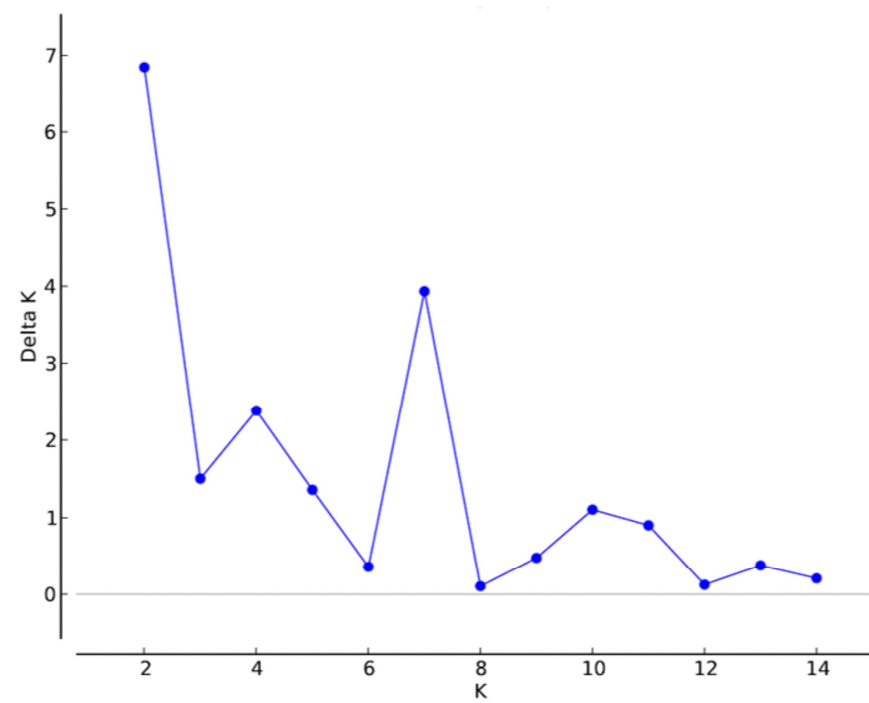

Supplement: S4 Fig — (a) Mean (± SD) log probability of the data [Ln Pr(XΙK)] over 10 runs, for each value of K. (b) ΔK values as a function of K, calculated according to Evanno et al. [100]. (PDF) [file pone.0277298.s004.pdf]

(a)

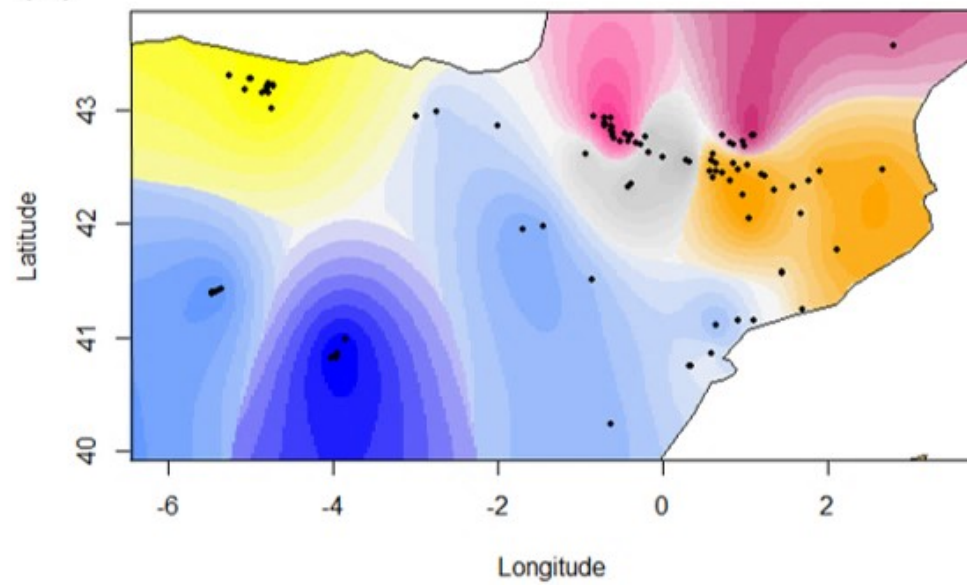

(b)

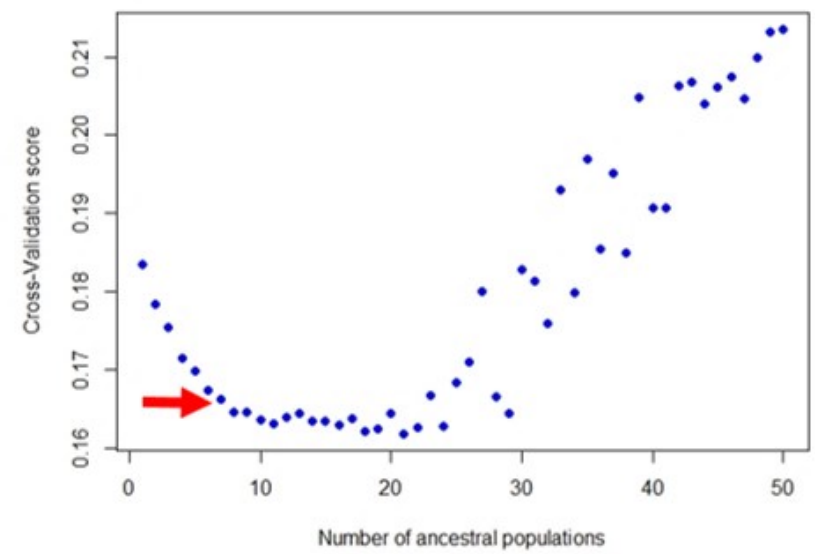

Supplement: S5 Fig — (a) Map depicting the distribution of ancestry coefficients inferred through Tess3R for K = 7 clusters. Black dots indicate the populations analysed. Colour codes as for STRUCTURE results. More saturated colours indicate a greater proportion of ancestry to either cluster. (b) Distribution of cross-validation scores according to the number of clusters. The red arrow indicates the number of clusters chosen for Tess3R analysis. (PDF) [file pone.0277298.s005.pdf]

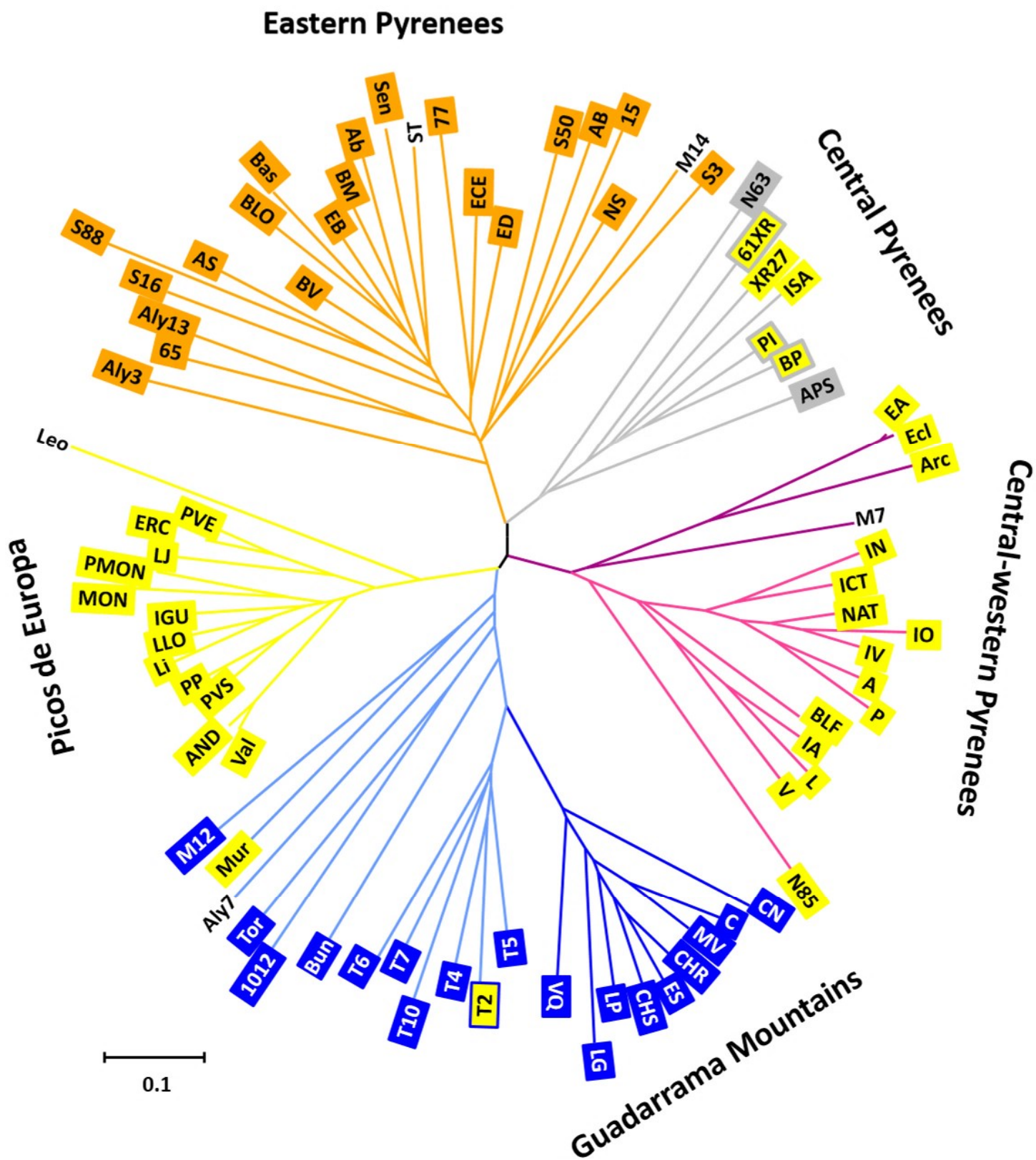

Supplement: S6 Fig — Branch colours delineate the seven genetic clusters inferred by STRUCTURE (see Figs 5A and 6), while colour shades around population codes correspond to the distinct mtDNA (ND4) haplogroups (blue: mtDNA haplogroup A, yellow: mtDNA haplogroup B, grey: mtDNA haplogroup E, orange: mtDNA haplogroup F; see Fig 3). In four populations (T2, 61XR, BP and PI) we detected the presence of more than one haplogroup. For population codes see S1 Table. (PDF) [file pone.0277298.s006.pdf]

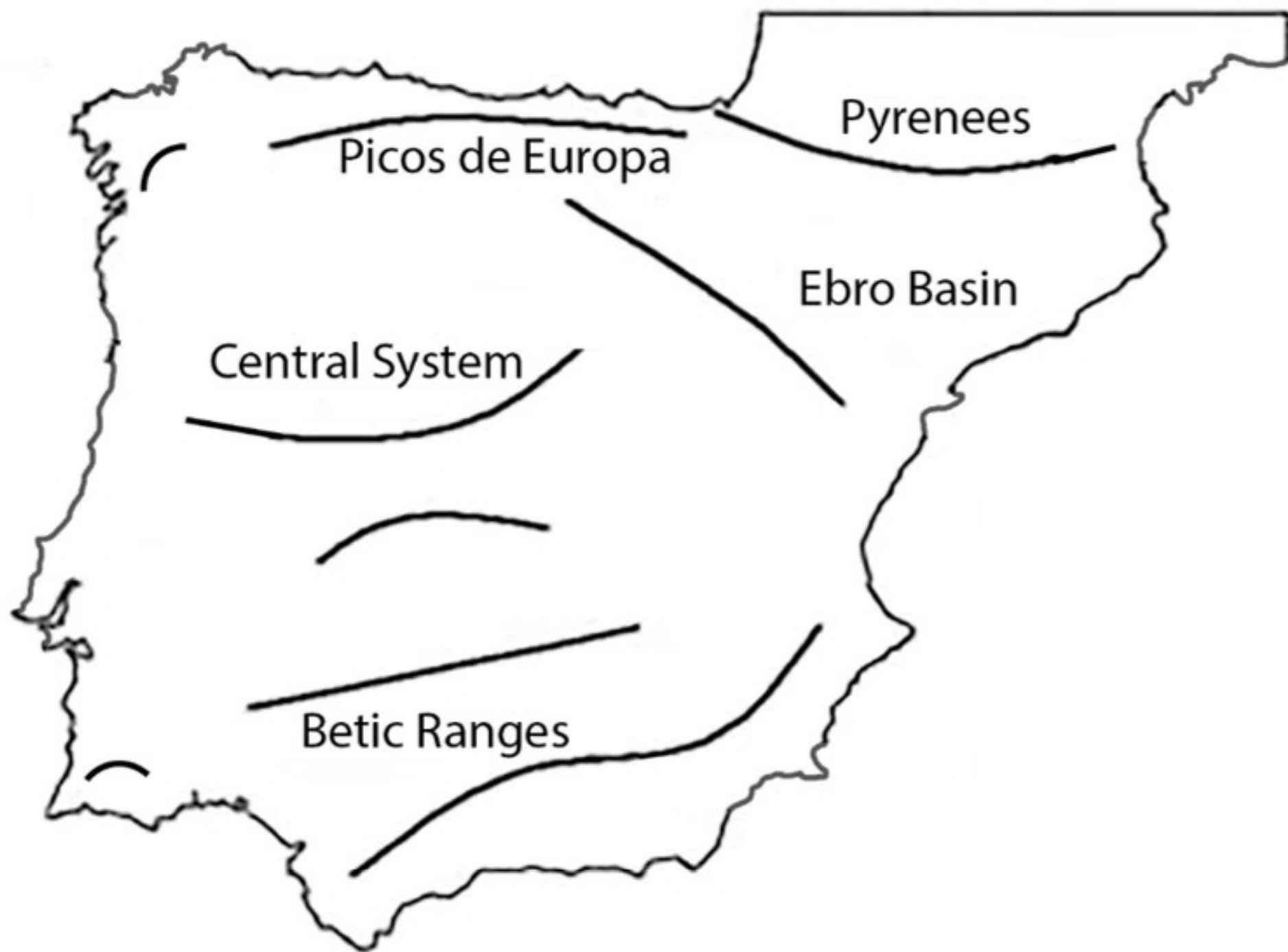

Supplement: S7 Fig — (PDF) [file pone.0277298.s007.pdf]
