# Supplementary material for: New insights on patterns of genetic admixture and phylogeographic history in Iberian high mountain populations of midwife toads
Source: PLoS One. 2022 Dec 1;17(12):e0277298. doi: 10.1371/journal.pone.0277298 (PMC9714896; doi:10.1371/journal.pone.0277298)
Supplement: S2 Table — The best supported scenarios were scenario 2 when considering only microsatellites (simple sequence repeats–SSRs) and scenario 5 when including both mtDNA (ND4) and microsatellite markers. See Fig 2 for more information on tested scenarios. N–effective population size for each analysed deme (EPY–eastern Pyrenees; CPY–central Pyrenees; CWPY–central-western Pyrenees; PEU–Picos de Europa Mountains, GUA–Guadarrama Mountains), ra–admixture rate, t–time of events in generations (t1 –time to the most recent split; t2 –time to the intermediate split; t3 –time to the most ancient split). Microsatellite (SSRs) and mitochondrial (ND4) parameters: mean μ–mean mutation rate, individual locus μ–individual locus mutation rate, mean P–mean coefficient P, individual locus P–individual locus coefficient P, SNI–Single Nucleotide Insertion rate, mean k–mean coefficient k, individual locus k–individual locus coefficient k. Microsatellite loci were divided in two groups depending on the motif length (tri- and tetranucleotide loci). Conditions: sequence data were simulated under a Tamura Nei (TN93) mutation model. (DOCX) [file pone.0277298.s009.docx]

**S2 Table. Parameters used in DIYABC analysis and respective priors for the best supported scenarios.** The best supported scenarios were scenario 2 when considering only microsatellites (simple sequence repeats – SSRs) and scenario 5 when including both mtDNA (ND4) and microsatellite markers. See Fig 2 for more information on tested scenarios.

|  | Microsatellites | | | | Microsatellites + ND4 | |  |
| --- | --- | --- | --- | --- | --- | --- | --- |
| Parameter | | Conditions | Distribution [min-max] | Conditions | | Distribution [min-max] | |
| N_EPY_ | |  | Uniform [10 - 20 000] |  | | Uniform [10 - 20 000] | |
| N_CPY_ | |  | Uniform [10 - 20 000] |  | | Uniform [10 - 20 000] | |
| N_CWPY_ | |  | Uniform [10 - 20 000] |  | | Uniform [10 - 20 000] | |
| N_PEU_ | |  | Uniform [10 - 20 000] |  | | Uniform [10 - 20 000] | |
| N_GUA_ | |  | Uniform [10 - 20 000] |  | | Uniform [10 - 20 000] | |
| N_EPY-GUA_ | |  | Uniform [10 - 40 000] |  | | - | |
| N_EPY-CWPY-GUA_ | |  | - |  | | Uniform [10 - 40 000] | |
| ra | |  | - |  | | 0.001 – 0.999 | |
| t_1_ | |  | Uniform [10 - 30 000] |  | | Uniform [10 - 30 000] | |
| t_2_ | | t_2_>t_1_ | Uniform [10 - 40 000] | t_2_>t_1_ | | Uniform [10 - 40 000] | |
| t_3_ | | t_3_>t_2_ | Uniform [10 - 50 000] | t_3_>t_2_ | | Uniform [10 - 50 000] | |
| Mean *µ1*_(SSRs)_ | |  | Uniform [10^-5^ - 10^-3^] |  | | Uniform [10^-5^ - 10^-3^] | |
| Individual locus *µ1*_(SSRs)_ | |  | Gamma [10^-5^ - 10^-2^] |  | | Gamma [10^-5^ - 10^-2^] | |
| Mean *P1*_(SSRs)_ | |  | Uniform [10^-1^ - 3x10^-1^] |  | | Uniform [10^-1^ - 3x10^-1^] | |
| Individual locus *P1*_(SSRs)_ | |  | Gamma [10^-2^ - 9x10^-1^] |  | | Gamma [10^-2^ - 9x10^-1^] | |
| SNI_(SSRs)_ | |  | Log-u [0] |  | | Log-u [0] | |
| Mean *µ2*_(SSRs)_ | |  | Uniform [10^-5^ - 10^-3^] |  | | Uniform [10^-5^ - 10^-3^] | |
| Individual locus *µ2*_(SSRs)_ | |  | Gamma [10^-5^ - 10^-2^] |  | | Gamma [10^-5^ - 10^-2^] | |
| Mean *P2*_(SSRs)_ | |  | Uniform [10^-1^ - 3x10^-1^] |  | | Uniform [10^-1^ - 3x10^-1^] | |
| Individual locus *P2*_(SSRs)_ | |  | Gamma [10^-2^ - 9x10^-1^] |  | | Gamma [10^-2^ - 9x10^-1^] | |
| SNI_(SSRs)_ | |  | Log-u [0] |  | | Log-u [0] | |
| Mean *µ*_(ND4)_ | | - | - | TN93 | | Uniform [10^-10^ - 10^-6^] | |
| Individual locus *µ*_(ND4)_ | | - | - | TN93 | | Gamma [10^-10^ - 10^-6^] | |
| Mean *k1*_(ND4)_ | | - | - | TN93 | | Uniform [0.05 - 20] | |
| Individual locus *k1*_(ND4)_ | | - | - | TN93 | | Gamma [0.05 - 20] | |
| Mean *k2*_(ND4)_ | | - | - | TN93 | | Uniform [0.05 - 20] | |
| Individual locus *k2*_(ND4)_ | | - | - | TN93 | | Gamma [0.05 - 20] | |

N – effective population size for each analysed deme (EPY – eastern Pyrenees; CPY – central Pyrenees; CWPY – central-western Pyrenees; PEU – Picos de Europa Mountains, GUA – Guadarrama Mountains), ra – admixture rate, t – time of events in generations (t_1_ – time to the most recent split; t_2_ – time to the intermediate split; t_3_ – time to the most ancient split). Microsatellite (SSRs) and mitochondrial (ND4) parameters: mean *µ* – mean mutation rate, individual locus *µ* – individual locus mutation rate, mean *P* – mean coefficient *P*, individual locus *P* – individual locus coefficient *P*, SNI – Single Nucleotide Insertion rate, mean *k* – mean coefficient *k*, individual locus *k* – individual locus coefficient *k*. Microsatellite loci were divided in two groups depending on the motif length (tri- and tetranucleotide loci). Conditions: sequence data were simulated under a Tamura Nei (TN93) mutation model.
