# Supplementary material for: New insights on patterns of genetic admixture and phylogeographic history in Iberian high mountain populations of midwife toads
Source: PLoS One. 2022 Dec 1;17(12):e0277298. doi: 10.1371/journal.pone.0277298 (PMC9714896; doi:10.1371/journal.pone.0277298)
Supplement: S4 Table — All P values < 0.001. (DOCX) [file pone.0277298.s011.docx]

**S4 Table. Analysis of molecular variance (AMOVA) for mitochondrial (ND4) and nuclear (microsatellites) markers based on the seven genetic groups identified by STRUCTURE in *Alytes obstetricans*/*almogavarii*** **(see Fig 6).** All P values < 0.001.

|  | ND4 |  |  | Microsatellites | |  |
| --- | --- | --- | --- | --- | --- | --- |
| Source of variation | SS | Variance component | % Variation | SS | Variance component | % Variation |
| Among clusters | 1149.248 | 6.259 | 84.945 | 1639.308 | 1.042 | 26.171 |
| Among populations within clusters | 148.165 | 0.466 | 6.319 | 1275.452 | 0.693 | 17.405 |
| Among individuals within populations | 77.883 | 0.644 | 8.736 | 3640.199 | 2.247 | 56.425 |
