# Supplementary material for: New insights on patterns of genetic admixture and phylogeographic history in Iberian high mountain populations of midwife toads
Source: PLoS One. 2022 Dec 1;17(12):e0277298. doi: 10.1371/journal.pone.0277298 (PMC9714896; doi:10.1371/journal.pone.0277298)
Supplement: S1 Appendix — (DOCX) [file pone.0277298.s014.docx]

**S1 Appendix. Supporting methods.**

**New insights on patterns of genetic admixture and phylogeographic history in Iberian high mountain populations of midwife toads**

Federica Lucati, Alexandre Miró, Jaime Bosch, Jenny Caner, Michael Joseph Jowers, Xavier Rivera, David Donaire-Barroso, Rui Rebelo and Marc Ventura

*Nuclear and mitochondrial genes sequencing*

Primers used for amplification and sequencing were: for cyt-*b*, a shortened version of primers Ptacek1-L (5’-TGAGGACAAATATCATTCTGAGG-3’) [1] and CB3Xen-H (5’-GGCGAATAGGAARTATCATTC-3’) [2]; for 12S, primers 12Sa [3] and H1557mod [4]; for 16S, primers 16sar and 16Sbr [5]. Amplification conditions followed standard PCR amplifications with annealing temperatures ranging between 48-56 ºC. As for ND4, primers ND4 and Leu, described by Arevalo et al. [6], were initially used for amplification and sequencing. PCR conditions followed Gonçalves et al. [7]. However, we obtained non-resolvable electropherograms and/or very short sequences for a high number of individuals. We thus designed a new set of primers specific for *A. obstetricans*/*almogavarii*, ND4-F (5’-TACCCCTTTATTGGTCTCGC-3’) and Leu-F (5’-GGTTCCTAAGACCAACGGAT-3’), which allowed us to recover high quality sequences. PCR conditions were the same as described in Gonçalves et al. [7], except for the annealing temperature that was set to 57 ºC. Similarly, we designed specific primers for β-fibint7 based on sequences that amplified using β-fibint7 primers BFxF and BFxR [8]. These primers were AlyFibF (5’-GACCTTATGATAATCATCTTTATTGGCT-3’) and AlyFibR (5’-TGTTTGTTTGGATCTGCATGTAGCCTGG-3’), with annealing temperatures ranging between 50-56 ºC. Primer combinations were: BFxF-BFxR, BFxF-AlyFibR and AlyFibF-BFxR.

*Genetic structure*

For microsatellites, population structure was inferred with a Discriminant Analysis of Principal Components (DAPC), using the ADEGENET package 2.1.1 [9, 10] in R 3.5.1 [11] (S1 Fig). The optimal number of clusters was assessed using the *find.clusters* function and determined as the K value above which BIC (Bayesian Information Criterion) values decreased substantially. We covered a range of possible clusters from 1 to 100 and retained all principal components (PCs), as suggested by Jombart and Collins [12]. We also used DAPC to investigate the hierarchical genetic structure of our data through the examination of various values of K and the sequence of differentiation when K increases. The same package was also used to perform a Principal Component Analysis (PCA), for comparison purposes. For DAPC analysis, 150 PCs (80% of variance) and all discriminant functions were retained. The number of PCs retained for DAPC was estimated using cross-validation through the *xvalDapc* command. Furthermore, the Bayesian cluster analysis implemented in STRUCTURE 2.3.4 [13] was performed to corroborate the optimal clustering solution inferred by DAPC and PCA analyses. All runs were repeated 10 times for each K, set between 1 and 15, with 100K burn-in steps followed by 100K MCMC repetitions. We used the admixture model with correlated allele frequencies. The optimal number of genetic clusters was determined using both the original method of Pritchard et al. [13] and the ΔK method of Evanno et al. [14], as implemented in STRUCTURE HARVESTER 0.6.94 [15]. The R package pophelper [16] was used to average replicate runs of the optimal K [17] and plot the final output. Genetic relationships between STRUCTURE clusters were visualised by constructing a neighbour-joining (NJ) tree based on net nucleotide distances [18] using the program NEIGHBOR in the PHYLIP package 3.695 [19]. In addition, to visualise genetic divergence between sampled sites and check for consistency with ND4-inferred lineages, we drew a NJ tree using the program POPTREEW [20]. We used Nei’s genetic distance (D_A_, [21]) and performed 1 000 bootstraps. As POPTREEW does not allow loci to have no data for an entire locality, we excluded 22 localities with data missing for at least one locus. Finally, we complemented previous analyses with a spatial-based clustering approach implemented in the R package Tess3R 1.1.0, a spatially explicit least-squares optimisation approach that incorporates geographic proximity information and a model-free algorithm [22]. Tess3R was run with 10 replicates for each of K=1-50 using default parameters, and the optimal K value was chosen using the cross-validation score as the value of K that corresponded to a plateau of the curve.

*Demographic history (DIYABC)*

To reduce computational demands, we selected 50 individuals from each of the five population groups, maximizing the number of samples with available ND4 sequences and minimizing missing data for microsatellites. The mutation rate prior distribution assumed for ND4 included a range of values comprehensive of the mutation rate calculated in a previous phylogenetic study (0.85 x 10^-8^ substitutions/site/year; [23]). We also performed a preliminary analysis run using a fixed prior for mutation rate that appeared unsuccessful, as the estimation of prior distribution of parameters showed a lack of correspondence between simulated and observed datasets (data not shown). The final parameter setting is shown in S2 Table. We generated 10^6^ simulated datasets per scenario, assuming a 1:1 female to male sex ratio and a generation time of 1 to 2 years [24]. The following summary statistics were used for microsatellites: mean number of alleles, mean genetic diversity and mean allele size variance as one sample statistics, and Fst and (dµ)² distance as pairwise statistics. As for ND4, the following summary statistics were used: number of segregating sites, mean of pairwise differences, variance of pairwise differences, Tajima’s D and private segregating sites as one sample statistics, and number of haplotypes and Fst as pairwise statistics. Pre-evaluation of scenarios, selection of the most supported scenario, confidence in scenario choice (type I and II errors), model checking, estimation of the posterior distribution of parameters and evaluation of bias and precision on parameters estimation for the most supported scenario followed Lucati et al. [25].

**References**

1. Ptacek MB, Gerhardt HC, Sage RD. Speciation by polyploidy in treefrogs: Multiple origins of the tetraploid, *Hyla versicolor*. Evolution. 1994;48(3):898-908.

2. Goebel A, Donnelly J, Atz M. PCR primers and amplification methods for 12S ribosomal DNA, the control region, cytochrome oxidase I, and cytochrome *b* in bufonids and other frogs, and an overview of PCR primers which have amplified DNA in amphibians successfully. Molecular Phylogenetics and Evolution. 1999;11(1):163-99.

3. Kocher TD, Thomas WK, Meyer A, Edwards SV, Pääbo S, Villablanca FX, et al. Dynamics of mitochondrial DNA evolution in animals: Amplification and sequencing with conserved primers. Proceedings of the National Academy of Sciences. 1989;86(16):6196-200.

4. Zaher H, Grazziotin FG, Cadle JE, Murphy RW, Moura-Leite JCd, Bonatto SL. Molecular phylogeny of advanced snakes (Serpentes, Caenophidia) with an emphasis on South American Xenodontines: A revised classification and descriptions of new taxa. Papéis Avulsos de Zoologia. 2009;49(11):115-53.

5. Palumbi S, Martin A, Romano S, McMillan WO, Stice L, Grabowski G. Simple fool's guide to PCR. Honolulu, Hawaii: University of Hawaii Press; 1991.

6. Arevalo E, Davis SK, Sites JW. Mitochondrial DNA sequence divergence and phylogenetic relationships among eight chromosome races of the *Sceloporus grammicus* complex (Phrynosomatidae) in Central Mexico. Systematic Biology. 1994;43(3):387-418.

7. Gonçalves H, Martínez-Solano I, Ferrand N, García-París M. Conflicting phylogenetic signal of nuclear vs mitochondrial DNA markers in midwife toads (Anura, Discoglossidae, *Alytes*): Deep coalescence or ancestral hybridization? Molecular Phylogenetics and Evolution. 2007;44(1):494-500.

8. Sequeira F, Ferrand N, Harris DJ. Assessing the phylogenetic signal of the nuclear β-Fibrinogen intron 7 in salamandrids (Amphibia: Salamandridae). Amphibia-Reptilia. 2006;27(3):409-18.

9. Jombart T, Devillard S, Balloux F. Discriminant analysis of principal components: A new method for the analysis of genetically structured populations. BMC Genetics. 2010;11(1):94.

10. Jombart T. *adegenet*: A R package for the multivariate analysis of genetic markers. Bioinformatics. 2008;24(11):1403-5.

11. R Core Team. R: A language and environment for statistical computing. Vienna, Austria: R Foundation for Statistical Computing. <http://www.R-project.org/>. 2018.

12. Jombart T, Collins C. A tutorial for discriminant analysis of principal components (DAPC) using *adegenet* 2.0.0. Imperial College London, MRC Centre for Outbreak Analysis and Modelling. 2015.

13. Pritchard JK, Stephens M, Donnelly P. Inference of population structure using multilocus genotype data. Genetics. 2000;155(2):945-59.

14. Evanno G, Regnaut S, Goudet J. Detecting the number of clusters of individuals using the software STRUCTURE: a simulation study. Molecular Ecology. 2005;14(8):2611-20.

15. Earl DA, vonHoldt BM. STRUCTURE HARVESTER: A website and program for visualizing STRUCTURE output and implementing the Evanno method. Conservation Genetics Resources. 2012;4(2):359-61.

16. Francis RM. pophelper: An R package and web app to analyse and visualize population structure. Molecular Ecology Resources. 2017;17(1):27-32.

17. Jakobsson M, Rosenberg NA. CLUMPP: A cluster matching and permutation program for dealing with label switching and multimodality in analysis of population structure. Bioinformatics. 2007;23(14):1801-6.

18. Pritchard JK, Wen X, Falush D. Documentation for STRUCTURE software, version 2.3. Department of Human Genetics University of Chicago, Department of Statistics University of Oxford. 2010.

19. Felsenstein J. PHYLIP (phylogeny inference package) version 3.6. Distributed by the author. Seattle (WA): Department of Genome Sciences, University of Washington. 2005.

20. Takezaki N, Nei M, Tamura K. POPTREEW: web version of POPTREE for constructing population trees from allele frequency data and computing some other quantities. Molecular Biology and Evolution. 2014;31(6):1622-4.

21. Nei M, Tajima F, Tateno Y. Accuracy of estimated phylogenetic trees from molecular data. Journal of Molecular Evolution. 1983;19(2):153-70.

22. Caye K, Jay F, Michel O, François O. Fast inference of individual admixture coefficients using geographic data. The Annals of Applied Statistics. 2018;12(1):586-608.

23. Gonçalves H, Maia-Carvalho B, Sousa-Neves T, Garcia-Paris M, Sequeira F, Ferrand N, et al. Multilocus phylogeography of the common midwife toad, *Alytes obstetricans* (Anura, Alytidae): Contrasting patterns of lineage diversification and genetic structure in the Iberian refugium. Molecular Phylogenetics and Evolution. 2015;93:363-79.

24. Tobler U, Garner TW, Schmidt BR. Genetic attributes of midwife toad (*Alytes obstetricans*) populations do not correlate with degree of species decline. Ecology and Evolution. 2013;3(9):2806-19.

25. Lucati F, Poignet M, Miró A, Trochet A, Aubret F, Barthe L, et al. Multiple glacial refugia and contemporary dispersal shape the genetic structure of an endemic amphibian from the Pyrenees. Molecular Ecology. 2020;29(15):2904-21.
